# Determining Risk Factors for Infection with Influenza A (H5N1)

**DOI:** 10.3201/eid1306.070025

**Published:** 2007-06

**Authors:** Janice Luisa Lukrafka, Alexandre Prehn Zavascki, Nêmora Barcellos, Sandra Costa Fuchs

**Affiliations:** * Universidade Federal do Rio Grande do Sul, Porto Alegre, Brazil

**Keywords:** Influenza A virus, H5N1 subtype, avian influenza, risk, statistical analysis, letter

## To the Editor

Novel antigenic subtypes of influenza viruses have been introduced periodically into the human population, resulting in large-scale global outbreaks (*1*). Highly pathogenic avian influenza (H5N1) viruses reemerged in 2003. Since then, they have reached endemic levels among poultry in several Southeast Asian countries, and across Asia, they have caused nearly 300 human infections, with a high rate of mortality (*1*,*2*). The results of many studies, including those for one recently conducted by Dinh et al. (*3*), have been published in an effort to identify the source(s) and modes of transmission of influenza A (H5N1) to humans and to guide the control and prevention of influenza infection.

Although new data regarding influenza A (H5N1) are urgently required, scientific rigor must be maintained during research and analysis to prevent misidentification of exposures as a risk factor for the disease and to prevent creation of iatrogenic panic among the exposed population and the scientific community (*4*). One point of scientific rigor that must be maintained is the use of adequate statistical analysis. The multivariate model in the study by Dinh et al. (*3*) was constructed by using a backward, stepwise variable selection strategy, in which variables with p<0.20 were included in the initial model*.* However, such a strategy has resulted in a first model and subsequent steps with far more than 10 variables per outcome (e.g., 28 persons with avian flu), resulting in model overfitting (i.e., a statistical model that is too complex for the amount of data), which could result in imprecise estimates or spurious associations (*5*).

We believe that scientific methods must be meticulously applied when planning, executing, analyzing, and interpreting the results of influenza (H5N1) studies to prevent identification of false risk factors for acquiring infection.

## References

[1] de Jong MD, Hien TT. Avian influenza A (H5N1). J Clin Virol. 2006;35:2–13. 10.1016/j.jcv.2005.09.002 16213784 PMC7108344

[2] World Health Organization. Epidemic and pandemic alert and response: confirmed human cases of avian influenza A (H5N1) [cited 2007 Apr 23]. Available from http://www.who.int/csr/disease/avian_influenza/country/en/index.html

[3] Dinh PN, Long HT, Tien NTK, Hien NT, Mai LTQ, Phong LH, Risk factors for human infection with avian influenza A H5N1, Vietnam, 2004. Emerg Infect Dis. 2006;12:1841–7. 17326934 10.3201/eid1212.060829 PMC3291373

[4] Bonneux L, van Damme W. An iatrogenic pandemic of panic. BMJ. 2006;332:786–8. 10.1136/bmj.332.7544.786 16575086 PMC1420757

[5] Concato J, Feinstein AR, Holford TR. The risk of determining risk with multivariable models. Ann Intern Med. 1993;118:201–10. 8417638 10.7326/0003-4819-118-3-199302010-00009

