# Supplementary material for: Determining Risk Factors for Infection with Influenza A (H5N1)
Source: Emerg Infect Dis. 2007 Jun;13(6):955–6. doi: 10.3201/eid1306.070025 (PMC2792835; doi:10.3201/eid1306.070025)
Supplement: Related texts

# Reply: In Response

**Authors:** Peter Horby

**Affiliations:** * National Institute for Infectious and Tropical Diseases, Hanoi, Vietnam

Lukrafka et al. (*1*) warn against the dangers of overfitting a regression model when the number of outcomes is <10 per variable, “which could result in imprecise estimates or spurious associations.” This warning is valid, but it is equally important to consider the relative merits of multiple analysis options given the data available, the difficulties in collecting the data, and the objective of the study. The objective of our study (*2*) was to explore possible risk factors for human infection with influenza A (H5N1) rather than to test an explicit a priori hypothesis or to obtain precise estimates of risk. We were limited to a finite number of cases, and had we slavishly followed criteria to avoid overfitting, we would not have run a regression model at all because we could have included only 2 variables, for which a stratified analysis would have been preferable. The regression model was run to confirm that the variables identified in the bivariate analysis retained their importance in the context of other variables; it was not intended to confirm or refute an a priori hypothesis, to be a predictive model, or to obtain precise and adjusted measures of risk. Despite the sample size limitations, we felt that looking at independence in a multivariable analysis was still valuable.

We explicitly acknowledge the limitations imposed by a small study size and were cautious in our interpretation, stating that the findings are the “basis for formulating new hypotheses.” The wide confidence intervals clearly indicate the low level of precision. The 3 variables in the final regression model were all statistically significant in bivariate analysis, and we do not believe they are spurious associations arising solely from an overfitted regression model.

## References

[1] Lukrafka JL, Zavascki AP, Barcellos N, Fuchs SC. Determining risk factors for infection with influenza A (H5N1) [letter]. Emerg Infect Dis. 2007;13:955–6.1758290910.3201/eid1306.070025PMC2792835

[2] Dinh PN, Long HT, Tien NTK, Hien NT, Mai LTQ, Phong LH, Risk factors for human infection with avian influenza A H5N1, Vietnam, 2004. Emerg Infect Dis. 2006;12:1841–7.1732693410.3201/eid1212.060829PMC3291373
